# Image-based phenomic prediction can provide valuable decision support in wheat breeding

**DOI:** 10.1007/s00122-023-04395-x

**Published:** 2023-06-27

**Authors:** Lukas Roth, Dario Fossati, Patrick Krähenbühl, Achim Walter, Andreas Hund

**Affiliations:** 1 Institute of Agricultural Sciences, ETH Zurich, Universitätstrasse 2, 8092 Zurich, Switzerland; 2 Agroscope, 1260 Nyon, Switzerland; 3 Delley Samen und Pflanzen AG, Route de Portalban 40, 1567 Delley, Switzerland

## Abstract

**Key message:**

**Genotype-by-environment interactions of secondary traits based on high-throughput field phenotyping are less complex than those of target traits, allowing for a phenomic selection in unreplicated early generation trials.**

**Abstract:**

Traditionally, breeders’ selection decisions in early generations are largely based on visual observations in the field. With the advent of affordable genome sequencing and high-throughput phenotyping technologies, enhancing breeders’ ratings with such information became attractive. In this research, it is hypothesized that G$\times$E interactions of secondary traits (i.e., growth dynamics’ traits) are less complex than those of related target traits (e.g., yield). Thus, phenomic selection (PS) may allow selecting for genotypes with beneficial response-pattern in a defined population of environments. A set of 45 winter wheat varieties was grown at 5 year-sites and analyzed with linear and factor-analytic (FA) mixed models to estimate G$\times$E interactions of secondary and target traits. The dynamic development of drone-derived plant height, leaf area and tiller density estimations was used to estimate the timing of key stages, quantities at defined time points and temperature dose–response curve parameters. Most of these secondary traits and grain protein content showed little G$\times$E interactions. In contrast, the modeling of G$\times$E for yield required a FA model with two factors. A trained PS model predicted overall yield performance, yield stability and grain protein content with correlations of 0.43, 0.30 and 0.34. While these accuracies are modest and do not outperform well-trained GS models, PS additionally provided insights into the physiological basis of target traits. An ideotype was identified that potentially avoids the negative pleiotropic effects between yield and protein content.

**Supplementary Information:**

The online version contains supplementary material available at 10.1007/s00122-023-04395-x.

## Introduction

Like natural evolution, plant breeding is driven by selection. Unlike nature, however, breeders are pressed for time: They have to achieve performance improvements within a few breeding generations, i.e., within a few years. Traditionally, many selection decisions are based on phenotypic observations combined with quality analyses. With the advent of ‘genomics’, these ‘breeders’ eye’ decisions became enhanced with genomic selection (GS) approaches (Meuwissen et al. 2001; Voss-Fels et al. 2019). GS promised to improve selection decisions, in particular for traits where the ‘breeders eye’ has a great potential to fail—e.g., in estimating yield potential in single row plots where harvest weight cannot be reliably determined (Rutkoski et al. 2016).

In parallel to genomics research, the field of ‘phenomics’ was growing, increasing both throughput and applicability of phenotyping technologies (Walter et al. 2015). These new phenotyping technologies are seen as key to bridge the gap between lab-determined genotypes and field-observation-based phenotypes (Crain et al. 2018). Consequently, secondary traits such as growth dynamics’ traits (Bustos-Korts et al. 2019; Millet et al. 2019; Diepenbrock et al. 2021) or disease resistance traits (Jia and Jannink 2012) were used as covariates in GS, enabling environment-specific predictions. Nevertheless, for small breeding companies, the entry hurdles for such phenotype-enhanced GS approaches are high, as they are faced with the challenge to simultaneously develop a genomics and phenomics workflow.

Alternatively, it was postulated that phenotyping has also the potential to directly enhance breeders’ decisions by means of ideotype concepts (Donald 1968). In ideotype-based breeding, one aims to construct a phenotypic ‘model’ (an ideal plant, thus ideotype) representing a unique combination of morphological and physiological attributes to optimize crop performance (Martre et al. 2015). Such a ‘phenomic selection’ (PS)—a selection process for a target trait purely based on (highly processed) secondary phenotypic traits^1^—could be particularly useful in early breeding stages where populations are large and plot sizes small (Rebetzke et al. 2019).

A common setup in breeding is the use of multi-environment trials (MET) where one can select for a wide adaption of target traits (Smith and Cullis 2018). In a recent study, we demonstrated that monitoring soybean METs with high-throughput field phenotyping (HTFP) can reveal strong relations between secondary traits and target traits, allowing to formulate an ideotypes concept (Roth et al. 2022a). Now, this knowledge is transferred to winter wheat (*Triticum aestivum* L.), while additionally including overall performance and stability considerations. It is hypothesized that genotype-by-environment (G$\times$E) interactions of secondary traits are less complex than those of the target trait. Under this premise, PS trained on MET data will allow selecting for overall performance and stability even in a single environment—i.e., an early-generation breeding nursery—assisted by high-throughput phenotyping.

A prerequisite of such an approach is the successful implementation of HTFP in METs and breeding experiments. The usefulness of HTFP in breeding is controversial—discussions mainly revolve around the contrast between what *can* be done in phenotyping (i.e., increasing the ‘stamp collection’ of traits) and what is *of value* for breeders (i.e., increasing genetic gain) (Rebetzke et al. 2019). A close interaction of breeders and ‘phenotypers’ is required to avoid the first and achieve the second. The ‘Trait spotting project’ as collaboration between a Swiss plant breeder (Delley Samen und Pflanzen/Agroscope) and the ETH Zurich as academic part aimed to foster such exchange. As part of the project, a drone-based phenotyping platform was developed (Roth et al. 2018b), and methods to extract plant height (Roth and Streit 2018), canopy cover/leaf area index (Roth et al. 2018a), and tiller count (Roth et al. 2020) for winter wheat were established. Furthermore, it was demonstrated that from these low-level traits one can derive three intermediate dynamics’ trait categories, timing of key stages (T), quantities at defined time points or periods (Q), and dose–response curves (C) (Roth et al. 2021, 2022c) (Table 1).

**Table 1:** Measured (low-level) traits and processed high-throughput field phenotyping (HTFP) traits of three categories, dose–response curve parameters (C), timing of key stages traits (T), and quantities traits (Q)

Trait category	Acronym	Description	Source
Low-level	PH	Plant height	Roth and Streit (2018)
	LA	Apparent leaf area	Roth et al. (2020)
	Tiller	Tiller per area	Roth et al. (2020)
C	$r_{{\text{max}}}$	Maximum growth rate at optimum temperature	Roth et al. (2022c)
	$T_{{\text{min}}}$	Base temperature of growth	Roth et al. (2022c)
	lrc	Responsiveness of growth to temperature increase	Roth et al. (2022c)
T	$t_{{\text{PH}}_{{\text{start}}}}$	Start of stem elongation	Roth et al. (2021)
	$t_{{\text{PH}}_{{\text{stop}}}}$	End of stem elongation	Roth et al. (2021)
	$t_{{\text{LA}}_{{\text{max}}}}$	Time point when apparent leaf area is maximized	Roth et al. (2022a)
	$t_{d{\text{LA}}_{{\text{max}}}}$	Time point when apparent leaf area increase is maximized	Roth et al. (2022a)
Q	${\text{PH}}_{{\text{max}}}$	Final height	Kronenberg et al. (2017)
	${\text{LA}}_{t{\text{PH}}_{15}}$	Apparent leaf area when plant height reaches 15%	Roth et al. (2022a)
	${\text{LA}}_{{\text{max}}}$	Maximum apparent leaf area	Roth et al. (2022a)
	$n_{{\text{tiller}}}$	Maximum number of tiller	Roth et al. (2020)

With this classification in mind, existing GS and PS literature for wheat focuses mainly on the first two categories. While Rutkoski et al. (2016) and Crain et al. (2018) combined GS with category Q traits (vegetation indices and canopy temperature measurements at defined growth phases) to predict yield, Sandhu et al. (2021) combined GS with Q traits (vegetation indices at defined time points) to both predict grain yield and protein content. Pure PS approaches are rare to find: Herrera et al. (2018) predicted yield on a spatial grid as result of environmental indices and phenology (Category T). Prey et al. (2020) used spectral indices to predict grain yield and confirmed a significant influence of the measurement time point (Category T).

While these results are of high value for crop physiologists and ecologists to broaden their research, they leave the main question of breeders unanswered: How well can these trait categories be used to complement the selection decision of a breeder, and in what way are they complementary to the ‘breeder’s eye’? This research aims to answer this question on the case example of a prediction model trained on several year-sites of a variety testing experiment and applied to a single-row breeding experiment.

## Materials and methods

### Field experiments

A variety testing experiment (‘Leistungspruefung’/LP01) was performed in two consecutive years at three sites in 2019 and two sites in 2020, resulting in a total of 5-year-site combinations. Respective sites were the field phenotyping platform site of ETH Zurich ‘FIP’ (Kirchgessner et al. 2017) (Lindau Eschikon; Switzerland; 47.449 N, 8.682 E; 556 m a.s.l., managed according to best practice in Swiss agriculture (‘ÖLN’)); the plant breeding site of Delley Samen und Pflanzen AG ‘Delley’ (Delley, Switzerland; 46.918 N, 6.979 E; 500 m a.s. l., extensively managed according to best practice for growth-regulator and fungicide-free Swiss agriculture (‘Extenso’)); and the testing site of the Strickhof competence center for food and agriculture ‘Strickhof’ (Lindau Eschikon; Switzerland; 47.445 N, 8.678, 530 m a.s.l., managed similar to ‘Delley’ (‘Extenso’)).

The experiments were part of the regular testing of advanced breeding material ($\ge$ F9) of Agroscope (Nyon, Switzerland)/Delley Samen und Pflanzen AG (Delley, Switzerland) and consisted of 45 elite winter wheat genotypes. Because of the variety testing character, the set for 2019 differed from the set for 2020 by nine genotypes, reducing the number of unique genotypes per year-site to 36.

Year-sites consisted of plots (experimental units in a row-range arrangement with spatial coordinates) enriched with block factors and genotypes. All year-sites contained four replications, except FIP 2020 where only three replications were used. Details about the experimental designs, soil, and management can be found in the Supplementary Materials. Meteorological data were obtained from a weather station next to the experimental field (50 m) for the FIP and Strickhof site and from a public Agrometeo weather station (http://www.agrometeo.ch/, Agroscope, Nyon, Switzerland) in proximity (800 m) for the Delley site. Air temperature was recorded 0.1 m above ground (FIP/Strickhof) and 0.05 m above ground (Delley) every 10 min and averaged per hour. Growing degree days (McMaster and Wilhelm 1997) for timing of key stage measurements were calculated assuming a base temperature of 0 $^\circ$C (Porter and Gawith 1999).

In 2020, an additional subset of 110 ears-to-row plots of F7 generation lines were monitored at Delley. This single ears’ descendants experiment was part of the last nursery trial before yield trials. For each line 20 head-to-rows were sown at Delley and, in parallel, 10 head-to-rows at Vouvry (Switzerland), a location with very high disease pressure (powdery mildew, yellow and brow rust, septoria and fusarium head blight). The combination of observations in both locations was used to choose the lines for the yield trials sown the next year. Around 250 lines were selected between approximately 1000 candidate’s lines.

In the subsequent year 2021, the selected lines from the F7 generation were cultivated as F8 generation yield plots. Yield was tested at four locations (Changins, Delley, Villars-le-Terroir, Ellighausen) across the Swiss Central Plateau, which can be considered the Swiss wheat belt. The experimental plots covered 7.1 m$^{2}$ (4.75 $\times$ 1.50 m) and consisted of eight rows with an inter-row distance of 0.16 m. Plots were separated by 1.3 m and sown at a rate of 350 seeds m$^{-2}$. Sowing and harvest took place during the months of October and June to July, respectively. The soils are mostly classified as Cambisols (World Resource Base, FAO). The mean value of four cultivars representing the leading cultivars for each Swiss quality class (TOP, 1 and 2), cv. ‘CH Nara’ (class TOP); cv ‘Montalbano’ (class TOP); cv ‘Hanswin’ (class 1); cv ‘Spontan’, (class 2), were used to calculate the relative yield values.

### Manual measurements

For the variety testing experiment, several manual measurements were performed. Beginning of stem elongation (GS30) was determined at 3 year-sites (Delley 2019, 2020, FIP 2019) in two replications by destructively measuring the distance between the basal node and the first extending node for three-to-five representative plants per time point and plot. When this distance reached ten millimeters, the plant was defined to be in the stem elongation stage (Zadoks et al. 1974). Heading (GS59) was defined as the time point, at which 50% of the spikes fully emerged from the flag leaf sheath (Meier 2018) and determined manually at 4 year-sites (Delley: 2019, 2020, FIP: 2019, 2020). Yield was determined by harvesting plots with a combine harvester (FIP and Strickhof: Nursery- master Elite; Delley: Classic; both Wintersteiger, Ried im Innkreis, Austria). Harvested seeds were dried at 30–35 $^\circ$C if necessary, and the harvest material pre-cleaned in a stand thresher and weighted. Humidity was determined using a HM-400 grain gauge (Harvest Data System, Wintersteiger GmbH, Ried, Austria). Yield was then arithmetically normalized to a water content of 15%. For the subsequent cleaning in an air separator and the estimation of protein content, plot samples of genotypes were merged to mixed probes. Protein content was estimated using a diode array NIR spectrometer [DA-7200, Perten Instruments (today PerkinElmer, Waltham, USA)].

**Fig. 1:**
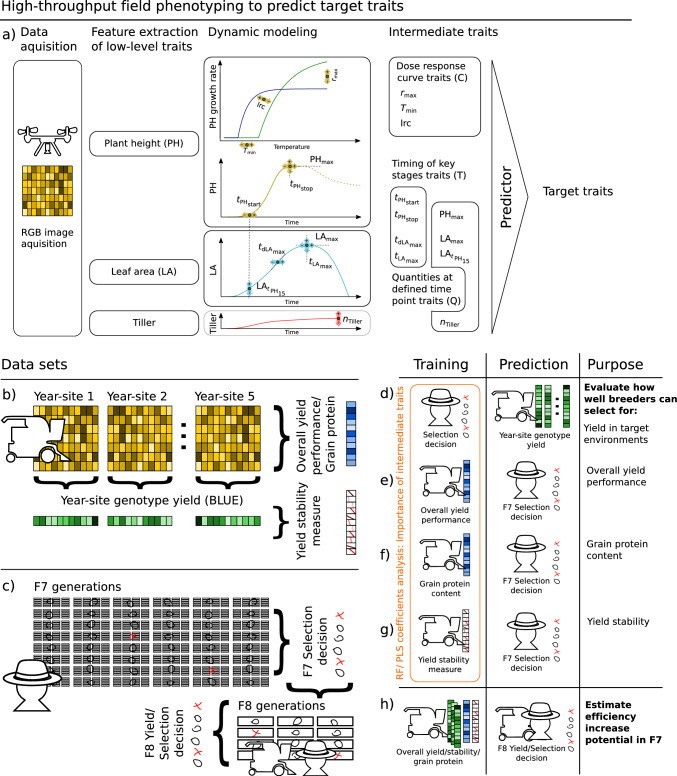
Workflow in high-throughput field phenotyping including data acquisition with drones, extraction of low-level traits, dynamic modeling, and predicting target traits (**a**), collected data for the variety testing (**b**) and F7/F8 breeding experiment (**c**), and how those datasets where used for training and prediction purposes (**d**–**h**)

The breeders’ selection notes for F7 lines were based on observations in the preliminary F6 generation in 2019 (one location, Changins) and the F7 lines described in the previous section in 2020 (two locations, Vouvry and Delley). In brief, these observations included in the F6 heading time, plant height, Zeleny indices, Thousand-Kernel-Weight (TKW), specific weight, grain hardness and (on a 1–9 scale), lodging, septoria tritici, leaf and stripe rust and grain appearance. In the F7, heading date, plant height, lodging resistance, disease resistance for leaf rust (2 notes) and stripe rust (3 notes) and Septoria tritici (1 note) were observed. The uniformity of the 20 head-to-rows in the F7 (Fig. 1c), stand density, and visual estimations of number of ears and length of ears were additional important criterion also taken into account for the selection.

### HTFP measurements

The variety testing experiment and the F7 generation experiment were both monitored with the unmanned aerial system (UAS) platform PhenoFly described in detail in Roth et al. (2020). The UAS captured RGB images with high spatial overlap that were processed using Structure-from-Motion software to digital elevation models and camera exposure positions. The flight height was 28 m, flight speed 1.8 m/s, percent end lap 92% and percent side lap 75%. Specific camera settings (e.g., exposure configurations) can be found in Roth et al. (2020). These settings led to a ground sampling distance of 3 mm, restricted motion blur to $\le$ 5%, and ensured a GCP recover frequency of > 70% for photos that showed one or more GCPs. For FIP 2019, 41 flights were performed between February 19 and July 10; for FIP 2020, 44 flights between February 12 and July 15; for Strickhof 2019, 20 flights between February 22 and July 8; for Delley 19, 21 flights between February 27 and July 12; for Delley 2020, 20 flights between April 6 and July 8.

After Structure-from-Motion processing, digital elevation models were further processed to plant height (PH) traits as described in Roth et al. (2018a). Apparent leaf area (LA) and tiller counts were extracted from processed multiview ground cover images as described in Roth et al. (2020). Based on these three low-level traits (PH, LA, and tiller counts) dynamics’ traits of the first two categories, T and Q were extracted according to Roth et al. (2021) using the P-spline/QMER method for LA and PH and using the GS30-based growth model method described in Roth et al. (2020) for tiller counts (Table 1). Dose–response curve parameters were extracted from PH measurements using high-frequency temperature measurements taken by the local weather station combined with lower-frequency drone-based plant height measurements to fit an asymptotic model (Roth et al. 2022c) (Fig. 1a). For an overview, all low-level and intermediate traits and corresponding literature references are listed in Table 1.

### Statistics

#### Adjusted genotype means per year-site and repeatability calculation

Intermediate traits of all three categories (C, T, Q) were processed in a stage-wise linear mixed model analysis, where the first stage averaged over within-year-site effects and the second stage over between-year-site effects (Roth et al. 2021). For the first stage, the R package SpATS (Rodríguez-Álvarez 2018) was parameterized with the model

1 $$\begin{aligned} \hat{\theta }_{{jk}} = {\theta }_{{ij}} + f(x(jk), y(jk)) + p_{r({{jk}})} + p_{c({{jk}})} + e_{{jk}}\,, \end{aligned}$$

where $i=(1, \dots , 45)$ is the *i*th genotype, $k=(1, \dots , 144)$ the *k*th plot, $j=(1, \dots , 5)$ the *j*th year-site, $\hat{\theta }_{{jk}}$ are plot responses based on dynamic modeling, ${\theta }_{ij}$ year-site genotype responses, $p_{c({jk})}$ range numbers of plots (main working direction, e.g., for sowing), $p_{r({jk})}$ row numbers of plots (orthogonal to main working direction), *f*(*x*(*jk*), *y*(*jk*)) a smooth bivariate surface in spatial *x* and *y* coordinates consisting of a bivariate polynomial and a smooth part (for details see Rodríguez-Álvarez 2018), and $e_{jk}$ plot residuals with ${\text{var}}(e) = \sigma ^2 w^{-1}$, while *w* are weights based on the standard error estimations from the previous dynamic modeling step, and $\sigma ^2$ the residual variance parameter. For best linear unbiased estimators (BLUEs) calculations, ${\theta }_{ij}$ was set as fixed, all other terms as random. For repeatability calculations, ${\theta }_{ij}$ was set as random. Within-year heritability (repeatability) was calculated according to Oakey et al. (2006).

#### Overall genotype means and heritability calculation

To calculate overall best linear unbiased predictors (BLUPs) and overall heritability for the 5 year-sites, a second stage of processing to overall adjusted genotype means (genotypic marginal means) was performed with the R package ASReml-R (Butler 2018) and the model

2 $$\begin{aligned}&\hat{\theta }_{{ij}} = \mu + u_{j} + {\theta }_{i} + (\theta u)_{ij} + e_{{ij}} \,, \end{aligned}$$

where $\hat{\theta }_{{ij}}$ are adjusted year-site genotype means (BLUE) from stage 1, $\mu$ a global intercept, $u_{j}$ year-site intercepts, ${{\theta }}_{{i}}$ genotype responses, $(\theta u)_{ij}$ genotype year-site interactions, and ${e}_{{ij}}$ residuals with ${\text{var}}(e)=\sigma ^2 w^{-1}$, where *w* are weights based on the diagonal of the variance–covariance matrix from the previous stage, and $\sigma ^2$ the residual variance parameter. $\mu$ and $u_{j}$ were set as fixed and all other terms as random. Heritability was calculated according to Cullis et al. (2006). Genotype year-site interactions $(\theta u)_{ij}$ were modeled using a heterogeneous variance model for year-sites (‘Diag’, allowing for both positive and negative variances), and alternatively using an uniform variance model (‘Id’). The model selection was then made based on the Bayesian information criterion (BIC).

#### Genetic correlation

For the genetic correlation calculation, the univariate model of Eq. 2 was extended to a bivariate model (Wright 1998; Holland et al. 2001),

3 $$\begin{aligned}&\left( \begin{array} {l} \hat{\theta _{{ij}}^{t1}} \\ \hat{\theta _{{ij}}^{t2}} \end{array} \right) = \left( \begin{array} {l} \mu ^{t1} \\ \mu ^{t2} \end{array} \right) + \left( \begin{array} {l} u^{t1}_j \\ u^{t2}_j \end{array} \right) \nonumber \\&\quad + \left( \begin{array} {l} \theta ^{t1}_{i} \\ \theta ^{t2}_{i} \end{array} \right) + \left( \begin{array} {l} (u\theta )_{ij}^{t1} \\ (u\theta )_{ij}^{t2} \end{array} \right) + \left( \begin{array} {l} {e}^{t1}_{{ij}} \\ {e}^{t2}_{{ij}} \end{array} \right) \,, \end{aligned}$$

where $\hat{\theta _{{ij}}^{t1}}$ and $\hat{\theta _{{ij}}^{t2}}$ are adjusted year-site genotype means (BLUEs) per trait (trait 1 ($^{t1}$) and trait 2 ($^{t2}$)), $\mu ^{t1}$ and $\mu ^{t2}$ global intercepts per trait, $u^{t1}_j$ and $u^{t2}_j$ year-site effects per trait, $\theta ^{t1}_{i}$ and $\theta ^{t2}_{i}$ genotype responses, and $(u\theta ^{t1})_{ij}$ and $(u\theta ^{t2})_{ij}$ the genotype responses to year-site interactions per trait. The terms $\mu ^{t1}$, $\mu ^{t2}$, $u^{t1}_j$ and $u^{t2}_j$ were set to fixed, all other terms to random. Note that *e* and $(u\theta )$ are confounded, wherefore the two terms were summarized in one variance-covariance structure. Genetic correlations among traits were then calculated based on the estimated variance and covariance components (Holland et al. 2001),

4 $$\begin{aligned} r_g = {\text{Corr}} ({\theta }^{t1}, {\theta }^{t2})={\frac{{\text{Cov}} ({\theta }^{t1} {\theta }^{t2}) }{{\sqrt{{\text{Var}} ( {\theta }^{t1} )}}{\sqrt{{\text{Var}} ( {\theta }^{t2} )}}}}\,. \end{aligned}$$

#### Factor-analytic mixed model for genotype year-site interaction analysis and overall performance and stability calculation

To analyze the severity of genotype year-site interactions for individual traits, Eq. 2 was extended with a factor-analytic (FA) component for year-site loadings and genotype scores based on Smith et al. (2001) with a reduced rank (RR) variance model (Thompson et al. 2003),

5 $$\begin{aligned}&\hat{\theta }_{{ij}} = \mu + u_j + {\text{RR}}(k_f)_{ij} + (\theta u)_{ij} + e_{{ij}} \,, \end{aligned}$$

where $k_f$ is the number of factors tested for, $\mu$ a global intercept, $u_{j}$ year-site intercepts, and $(\theta u)_{ij}$ lack-of-fit effects for genotype year-site interactions modeled using a heterogeneous variance model for year-sites (Diag). This FA model was applied in ASReml-R to all intermediate traits and to the target traits yield and grain protein content. The 5 year-sites used in this study allowed for a maximum of two factors for the FA model, $k_f = (1, 2)$. Note that instead of the one-stage approach proposed by Smith et al. (2001), a two-stage approach based on year-site BLUEs (Eq. 1) was used with residuals *e* set to ${\text{var}}(e) = \sigma ^2 w^{-1}$, where *w* are weights based on the diagonal of the variance–covariance matrix from Stage 1, and $\sigma ^2$ the residual variance parameter (Piepho et al. 2012).

To determine the complexity of G$\times$E interactions, two models differing only in the number of factors (FA1–FA2) and two based on Eq. 1 were fitted to data, and their performance compared based on the Bayesian information criterion (BIC). The BIC was calculated using the full log-likelihoods (Verbyla 2019) with the function *icREML* provided therein. The BIC can be seen as an advancement of the Akaike information criterion (AIC) that includes a reduction of effective sampling size caused by the dependence of the response (Müller et al. 2013). For intermediate traits, traits where the BIC suggested complex G$\times$E interactions—i.e., FA1 or FA2 models—were discarded from further analysis. This affected the traits $\text{LA}_{t_{\text{PH}_{15}}}$ and $\text{LA}_\text{max}$. As the trait $t_{\text{LA}_\text{max}}$ is based on $\text{LA}_\text{max}$, it was excluded as well.

For target traits, discarding traits with complex G$\times$E interactions was not an option, as predicting both target traits was among the aims of this work. Therefore, if the BIC suggested a simple linear mixed model without G$\times$E (Id), this model was selected, but if any other model was suggested, the FA2 model was preferred, as it allows a dissection in stability and overall performance of the target trait. Effectively, this was the case for yield but not for grain protein content.

To estimate the corresponding genotype yield performance and stability across year-sites, the FAST approach as described in Smith and Cullis (2018) was chosen that extracts an overall yield performance (OP) indicator and a stability measure (root-mean-squared deviation, RMSD). Beforehand, the estimates of loadings were rotated as proposed by Smith and Cullis (2018) using the R-code provided in Smith et al. (2021).

#### Target trait prediction

To predict yield and grain protein content, two approaches were tested: Partial least squares regression (PLS) and random forest regression (RF). PLS uses a linear multivariate model to relate a matrix of observable variables $\varvec{X}$ to a matrix of responses $\varvec{Y}$ (Wold et al. 2001). The underlying assumption of correlations among $\varvec{X}$ allows PLS to analyze noisy and co-linear data, which makes it particularly useful for HTFP. RF on the other hand is an ensemble learning method that combines multiple decision trees to one model, making it similarly suited for highly correlated HTFP data but additionally allowing nonlinear mappings of $\varvec{X}$ to $\varvec{Y}$. PLS was fitted in R (R Core Team 2019) with the package *pls* (Liland et al. 2021), RF with the package *ranger* (Wright and Ziegler 2017).

To compare the performance of the two algorithms (PLS and RF), year-site BLUEs of all three categories (C, T, Q) were used as predictors for year-site BLUEs of yield (Fig. 1a, c). Cross-validation (CV) included three different resampling setups: (1) Unseen environments (unseen E): fivefold CV with each fold excluding a whole year-site for the training set that is used as test set, (2) Unseen genotypes (unseen G): 45-fold CV with each fold excluding a genotype for the training set that is used as test set, (3) Unseen genotypes and unseen environments (unseen G and E): 179-fold CV with each fold excluding a complete year-site and a complete genotype while the excluded genotype in the excluded year-site is used for the test set. For RF, hyperparameter tuning was performed for each resampling step using grid-search in a tenfold CV for unseen E and unseen G and in a leave-one-out CV for unseen G and E. Tuned parameters included the number of trees (ntree, lower: 100, upper: 1000) and the number of variables randomly sampled at each split (mtry, lower: 1, upper: 11). For PLS, the number of components (ncomp) was determined beforehand using all data points in a tenfold CV per trait.

Both algorithms were then taken to compare the suitability of the three trait categories as predictors for OP (Fig. 1a, d), yield stability (RMSD) (Fig. 1a, g), and selection decision (Fig. 1b, e). Models were trained four times: for PLS with timing traits only (*f*(*T*)), with quantities at defined time points only (*f*(*Q*)), with dose–response curve parameters only (*f*(*C*)), and for PSL as well as RF with all three trait categories (*f*(*T*, *Q*, *C*)). The importance of features for PLS was extracted based on the summarized scores for all components per features, and for RF based on 50-fold permutation runs per feature.

For the breeders’ selection decision dataset, the response is, in contrast to yield, not continuous but categorical. Hence, the selection scale (line selected, sister line selected, line repeated in next generation, sister line repeated in next generation, discarded) was transformed to an ordinal scale (3, 2, 1, 0, − 1) that was then treated as regression problem.

Before model fitting, one outlier was removed for the yield dataset (LP01) with $n_\text{tiller}>8000$. For the breeders’ selection decision dataset, 354 out of 2200 rows had to be removed because the dose–response curve fitting failed to converge, resulting in missing values for $r_\text{max}$, $T_\text{min}$ and lrc. As optimization metric for PLS and RF, the root-mean-square error (RMSE) was used, and additionally the normalized RMSE (nRMSE) and Spearman’s rank correlation coefficient ($r_\textrm{s}$) provided.

#### Efficiency of selection

To compare a pure breeders’ selection decision approach with a HTFP-enriched selection decision approach, a HTFP overall yield performance prediction-based threshold was evaluated. This threshold was applied to the already performed selection in the F8 generation experiment. The efficiency of selection was then calculated as the ratio between selected lines and total number of lines with and without applying an additional HTFP threshold to breeders’ decisions. Subsequently, the increase in efficiency was derived as the difference between breeders’ selection-based efficiency and HTFP-enriched efficiency.

## Results

### Modeling the dynamic of low-level traits enables to extract heritable but partly correlated intermediate traits

Time series of the low-level trait plant height (PH) indicated a clear start of growth early in the season, followed by a close-to-linear increase phase and a stagnation at a final height afterward (Fig. S1a). While the extracted timing of key stages traits $t_{\text{PH}_{\text{start}}}$ and $t_{\text{PH}_{\text{stop}}}$ were detected for all plots of the variety testing trials, for the F7 generation breeding trial, the sparse flight density in the early season prevented the extraction of $t_{\text{PH}_{\text{start}}}$ for some plots (Fig. S1a, ‘Delley (C), 2020’). The quantity trait $\text{PH}_\text{max}$ was reliably detected for all plots.

Time series of the low-level trait apparent leaf area (LA) showed higher fluctuations between time points and year-sites than PH time series, but also a clear start of growth followed by an exponential growth phase and a decrease phase with high fluctuations afterward (Fig. S1b). Despite that for some plots two peaks of LA were modeled by the P-spline, visual inspections of plots confirmed that the timing trait $t_{\text{LA}_{\text{max}}}$ and quantity trait $\text{LA}_\text{max}$ were reliably allocated to the first peak. Consistently, the time point of maximum LA growth $t_{d{\text{LA}_{\text{max}}}}$ was detected in the increase phase before the first peak (Fig. S1b, ‘FIP, 2019’, ‘Strickhof, 2019’). Unlike $t_{d{\text{LA}_{\text{max}}}}$, the quantity trait $\text{LA}_{t{\text{PH}_{15}}}$ was extracted on a wide range between the start of LA increase and $t_{\text{LA}_{\text{max}}}$.

The extracted dose––response curve parameters for the temperature response of stem elongation showed a large spread of ${T}_\text{min}$ and $r_\text{max}$ (Fig. S1c). While some plots showed almost binary and very unresponsive shapes (Fig. S1c, ‘Delley, 2019’), the curves of others appeared more curvy and hence indicated a stronger responsiveness to temperature (Fig. S1c, ‘Strickhof, 2019’).

Calculating year-site specific repeatabilities for all intermediate traits revealed strong variations between year-sites for certain traits (Table 2), but no clear trait-independent systematic effect of year-sites. For example, for Delley 2019, the trait lrc showed a below-average repeatability, while other traits were not affected. When comparing HTFP traits with manual measurements, the growth stage ratings GS30 and GS59 showed similar strong variations in repeatability. In contrast, yield repeatability was constant and high for all year-sites. Repeatability values for grain protein content are not available as the measurements were done on mixed probes per genotype.

The highest overall heritability was calculated for the growth stage rating GS59, followed by grain protein content and yield (Table 2). For HTFP traits, the heritability varied from very low values (${\text{LA}_{\text{max}}}$) to very high values ($\text{PH}_\text{max}$). Notably, the heritabilities of timing of key stage traits all settled in the middle range ($h^2=0.52-0.71$), while the ones for quantities and dose–response curve traits were at the upper and lower extremes.

**Table 2:** Repeatabilities (regular) and heritabilities (bold) for intermediate high-throughput field phenotyping (HTFP) traits of the three categories dose–response curve parameters (C), timing of key stages traits (T), and quantities traits (Q) and manually measured growth stage (GS) traits, yield and grain protein content

Category	Parameter	Delley, 2019	Delley, 2020	FIP, 2019	FIP, 2020	Strickhof, 2019	(All)
C	$T_{\text{min}}$	0.77	0.82	0.41	0.37	0.72	**0**.**49**
	lrc	0.18	0.82	0.22	0.25	0.57	**0**.**84**
	$r_{\text{max}}$	0.75	0.53	0.26	0.39	0.78	**0**.**44**
T	$t_{{\text{PH}}_{\text{start}}}$	0.44	0.32	0.70	0.01	0.91	**0**.**71**
	$t_{{\text{PH}}_{\text{stop}}}$	0.44	0.38	0.71	0.39	0.80	**0**.**66**
	$t_{d{\text{LA}}_{\text{max}}}$	0.72	0.30	0.73	0.73	0.59	**0**.**56**
	$t_{{\text{LA}}_{\text{max}}}$	0.77	0.66	0.85	0.82	0.74	**0**.**52**
Q	${\text{PH}}_{\text{max}}$	0.77	0.62	0.79	0.49	0.94	**0**.**91**
	${\text{LA}}_{\text{max}}$	0.83	0.76	0.93	0.92	0.72	**0**.**36**
	$n_{\text{tiller}}$	0.78	0.27	0.73	0.50	0.77	**0**.**41**
	${\text{LA}}_{t{\text{PH}}_{15}}$	0.84	0.54	0.94	0.83	0.85	**0**.**60**
Manual	GS30	0.89	0.44	0.71	–	–	**0**.**90**
	GS59	0.98	0.84	0.45	0.89	–	**0**.**97**
	Yield	0.92	0.94	0.94	0.93	0.95	**0**.**92**
	Grain protein	–	–	–	–	–	**0**.**93**

**Fig. 2:**
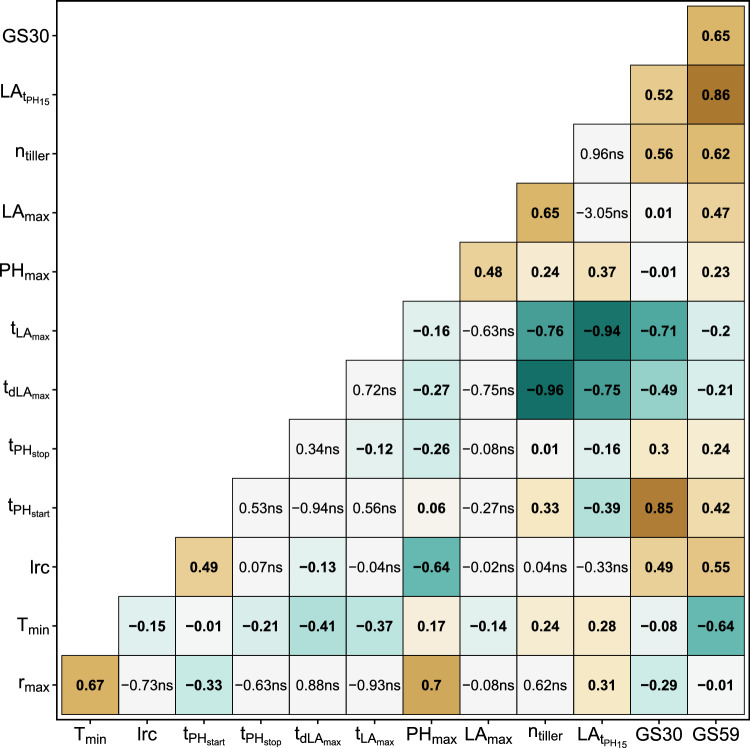
Genetic correlations of year-site BLUEs for HTFP traits and manual growth stage (GS) measurements

When calculating genetic correlations, clear relations between HTFP traits and growth stage ratings became visible (Fig. 2): For GS59, a very strong positive correlation was found for ${\text{LA}}_{t{\text{PH}}_{15}}$ and a strong correlation for $n_{\text{tiller}}$, indicating that the apparent leaf area in an early growth stage—which was highly (but not significantly) correlated to the number of tillers—is related to the time point of heading. For GS30, a very strong positive correlation to $t_{{\text{PH}}_{\text{start}}}$ was found, confirming the suitability of $t_{{\text{PH}}_{\text{start}}}$ as proxy trait to determine jointing. The partial interdependence of GS30 and GS59 was confirmed by a strong correlation between them.

In addition to the previously mentioned relations, a high negative correlation between GS30 and $t_{{\text{LA}}_{{\text{max}}}}$ was found, indicating a trade-off between early growth and apparent leaf area mid-season. Nevertheless, a vigorous early growth also led to an early mid-season development, indicated by the strong negative correlation between $t_{{\text{LA}}_{{\text{max}}}}$ and $n_{\text{tiller}}$, by the very strong negative correlation between $t_{{\text{LA}}_{{\text{max}}}}$ and ${\text{LA}}_{t{\text{PH}}_{15}}$, and by the strong negative correlation between $t_{d{\text{LA}}_{\text{max}}}$ and ${\text{LA}}_{t{\text{PH}}_{15}}$. Interestingly, $t_{{\text{LA}}_{{\text{max}}}}$ was also negatively correlated with the dose–response curve parameter $T_{\text{min}}$, indicating that high base temperatures of growth are related to an early time point where the maximum apparent leaf area is reached. This finding was further confirmed by the strong negative correlation between $T_{\text{min}}$ and GS59.

The strongest relationship was found between $t_{d{\text{LA}}_{\text{max}}}$ and $n_{\text{tiller}}$. In addition, $n_{\text{tiller}}$ was strongly positive correlated to ${\text{LA}}_{\text{max}}$. Consequently, high numbers of tillers were associated with an early rapid development of apparent leaf area and large leaf area mid-season.

For the three dose–response curve traits, $T_{\text{min}}$ and $r_{\text{max}}$ were strongly correlated. While $r_{\text{max}}$ and lrc had a strong correlation to ${\text{PH}}_{\text{max}}$, the correlation of $T_{\text{min}}$ with ${\text{PH}}_{\text{max}}$ was low, indicating that final height was mainly driven by growth at optimum temperature and steepness of the temperature response. All other relations between HTFP traits showed either only moderate of low correlations or were not significant.

### G$\times$E interactions for yield are complex, but less complex for HTFP traits and grain protein content

**Fig. 3:**
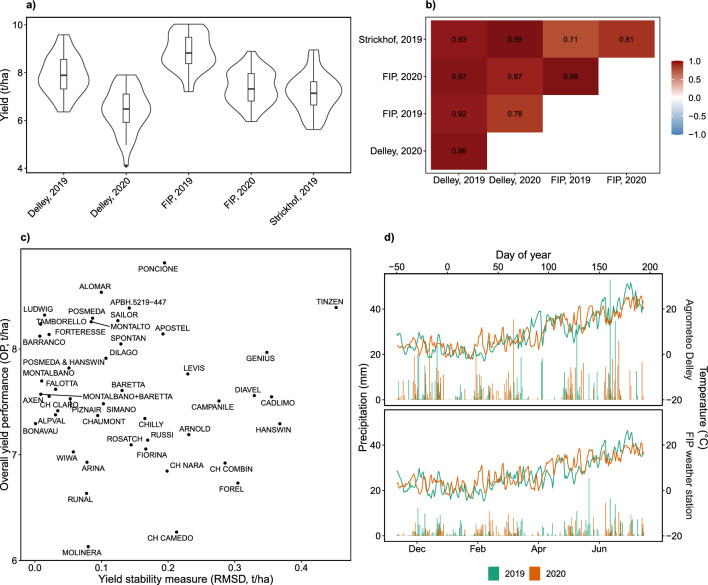
Yield performance of genotypes as BLUEs for individual year-sites (**a**), estimated genetic correlation for common genotype by environment interactions (**b**), overall performance versus stability based on a factor-analytic mixed model (FA2) (**c**), and characterization of sites with temperature and precipitation for 2019 and 2020 (**d**)

The courses of meteorological covariates (temperature and precipitation) showed large differences between years and smaller differences between sites (Fig. 3d). 2019 was characterized by frequent rainfall throughout the season, but 2020 was characterized by a dry phase between beginning and end of April. Precipitation for the Delley site was higher than for the FIP/Strickhof sites. Temperature courses for all sites were very comparable, but differed between years, with hotter periods toward the end of the season in 2020 than in 2019. Consequently, genotype yields for year-sites (BLUEs) varied between year-sites with lowest yields for Delley, 2020 and highest yields for FIP, 2019 (Fig. 3a).

Yield was further examined using a factor-analytic model with two factors (FA2). Estimated genetic correlations for common G$\times$E interactions revealed that while some year-sites were highly related (e.g., Strickhof, 2019 and Delley, 2020), others had lower correlations (e.g., FIP, 2019 and Strickhof, 2019) (Fig. 3b). Calculating overall yield performance (OP) and yield stability (RMSD) using the FAST approach revealed that the examined genotypes covered well the range of measured OP but clustered at low RMSD (corresponding to high yield stability) with a few outliers with high RMSD (corresponding to low yield stability) (Fig. 3c).

For grain protein content, a simple linear mixed model with uniform variance for G$\times$E interactions was suggested by BIC, indicating only small G$\times$E effects in the examined environments (Table 3). Consequently, no dissection in stability and overall performance was performed.

For intermediate traits, model selection was performed based on BIC following the strategy described in Sect. 2.4.4. For most traits, linear mixed models showed better BICs than factor-analytic models, indicating non-complex G$\times$E interactions (Table 3). Nevertheless, for the two quantity traits ${\text{LA}}_{t_{{\text{PH}}_{15}}}$ and ${\text{LA}}_{\text{max}}$, factor-analytic models with one factor (FA1) performed best. Consequently, those to traits were excluded from further analysis. For ${\text{LA}}_{\text{max}}$, a FA1 model with mixed positive and negative loadings was suggested, indicating crossover G$\times$E interactions. Consequently, the timing trait $t_{{\text{LA}}_{\text{max}}}$ that is derived from ${\text{LA}}_{\text{max}}$ was excluded as well.

**Table 3:** BIC and full log-likelihood for two linear mixed models with different variance components (Id and Diag) and two factor-analytic models with increasing number of factors (FA1–FA2)

Category	C			T				Q				Target trait	
Trait	$r_{\text{max}}$	$T_{\text{min}}$	lrc	$t_{{\text{PH}}_{\text{start}}}$	$t_{{\text{PH}}_{\text{stop}}}$	$t_{d{\text{LA}}_{\text{max}}}$		${\text{PH}}_{\text{max}}$		$n_{\text{tiller}}$		Protein	Yield
*Model BIC*													
Id	− 635	339	***397***	***1309***	***1450***	1490	*1741*	***− 1076***	− 779	***2363***	− 809	***85***	51
Diag	***− 662***	***328***	412	1313	1461	***1486***	1755	(–)	*− 795*	2366	(–)	(–)	*45*
FA1	*− 651	*342	*419	*1322	1467	1507	*1760	− 1066	*− 795	2375	*− 845*	104	53
FA2	*− 642	*357	*432	*1338	1481	*1521	1774	− 1061	*− 788	*2375	*− 839	120	**56**
*Model Log-likelihood*													
Id	335.5	− 151.7	− 177.9	− 634	− 704.3	− 727.1	− 852.5	559	410.3	− 1163.1	422.8	− 24.4	− 4.7
Diag	362.2	− 135.8	− 175.2	− 630.7	− 701.7	− 719.4	− 846.5	(–)	413	− 1151.9	(–)	(–)	8.5
FA1	364.3	− 137.5	− 173.1	− 629.7	− 694.7	− 719.8	− 841	574.6	438.9	− 1146	461.3	− 23.3	9.9
FA2	367.3	− 137.1	− 172.2	− 627.6	− 693.7	− 719	− 840	579.7	440.5	− 1143.4	466.2	− 20.9	16

Models that did not converge are marked with (–). Lowest BIC values per intermediate trait are marked in italic, the selected model in bold. Discarded traits are marked with strikeout and reasons indicated in underline

*Indicate factor-analytic models where the first factor has mixed positive and negative loadings

### Partial least square as yield predictor shows less tendency to overfitting than random forest

The performance of predictors for genotype BLUEs with traits of all three categories (C, T, Q) varied strongly depending on the resampling strategy (Table 4). In the most advantageous situation where only the genotype was unseen, RF outperformed PLS with strong correlations and the lowest RMSE and nRMSE. Nevertheless, when using unseen environments as resampling strategy, the correlation dropped for both algorithms, indicating severe overfitting. Further intensifying the resampling strategy to unseen genotypes in unseen environments restored the prediction capacity to some extent for PLS, but less for RF.

Consequently, PLS was favored for the target traits overall yield performance, yield stability, grain protein content and breeders’ selection decisions. The hyperparameter of PLS (number of components; ncomp) was re-tuned for each trait. This resulted in a PLS with one component (PLS(1)) for overall yield performance, stability, and grain protein content, and a PLS with four components (PLS(4)) for selection decision predictions.

**Table 4:** Spearman’s rank correlation ($r_s$), root mean squared error (RMSE) and normalized RMSE (nRMSE) of benchmarks with partial least square (PLS) and random forest (RF) regressors for different resampling strategies and combinations of timing of key stage (T), quantities (Q) and dose–response curve parameter (C) predictors

Task	Learner	Resampling	$r_s$	RMSE	nRMSE
Yield, Year-site BLUEs				(t/ha)	(%)
f(T, Q, C)	PLS (1)	Unseen E	0.17	1.23	20.80
f(T, Q, C)	RF	Unseen E	− 0.05	1.36	22.90
f(T, Q, C)	PLS (1)	Unseen G	0.59	0.88	14.80
f(T, Q, C)	RF	Unseen G	0.89	0.73	12.40
f(T, Q, C)	PLS (1)	Unseen G and E		1.06	17.80
f(T, Q, C)	RF	Unseen G and E		1.09	18.30
Overall yield performance (OP)				(t/ha)	(%)
f(T, Q, C)	PLS (1)	Eightfold rep. cv	0.43	0.49	18.20
f(C)	PLS (1)	Eightfold rep. cv	0.32	0.57	21.20
f(Q)	PLS (1)	Eightfold rep. cv	0.40	0.50	18.70
f(T)	PLS (1)	Eightfold rep. cv	0.43	0.52	19.30
Grain protein content				(%)	(%)
f(T, Q, C)	PLS (1)	Eightfold rep. cv	0.27	0.74	23.90
f(C)	PLS (1)	Eightfold rep. cv	0.03	0.81	26.00
f(Q)	PLS (1)	Eightfold rep. cv	0.34	0.72	23.20
f(T)	PLS (1)	Eightfold rep. cv	0.27	0.75	24.10
Yield stability (RMSD)				(t/ha)	(%)
f(T, Q, C)	PLS (1)	Eightfold rep. cv	0.01	0.12	26.30
f(T, Q, C)	RF	Eightfold rep. cv	0.30	0.11	23.50
Selection decision, F7				(–)	(%)
f(T, Q, C)	PLS (4)	Tenfold rep. cv	0.26	1.21	30.30

### Quantity traits are good protein content predictors, overall performance and yield stability predictions profit from traits of all three categories

For overall yield performance (OP) predictions, quantity traits (Q) and timing traits (T) contributed the most to both a high prediction accuracy ($r_s$) and low RMSE (Table 4). Using only dose–response curve traits (C) resulted in poorer predictors. Nevertheless, combining traits of all three categories (C, T, Q) could further improve the RMSE in comparison with using only Q or T traits.

For grain protein content, Q traits contributed the most to a high accuracy and low RMSE. Using only T traits resulted in a slightly lower correlations to grain protein content, using only C traits in a very poor prediction accuracy. In opposite to yield, the performance of a grain protein content predictor that combines traits of all three categories (C, T, Q) was slightly lower than that of a predictor purely based on Q traits. Nevertheless, the combined predictor was favored for further analysis to allow a comparison of feature scores with other target trait predictors.

For yield stability (RMSD), the prediction performance using PLS was inferior to the accuracy for other traits. Therefore, RF was reconsidered and could indeed restore the prediction accuracy to some extent, indicating that nonlinear combinations of traits of all three categories were required to predict stability.

### Breeders select for overall performance and yield stability but select against grain protein content

**Fig. 4:**
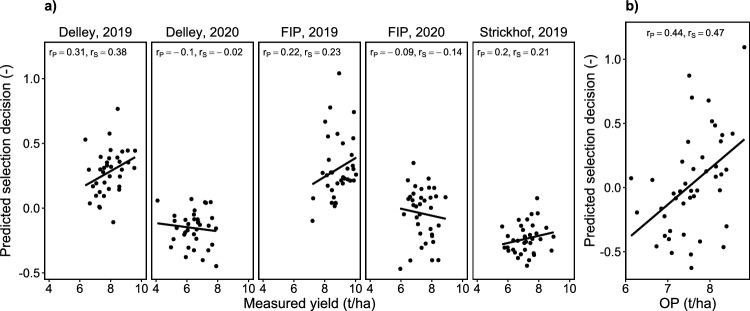
Prediction of selection decision for individual year-sites versus measured yield (**a**) and prediction of selection decision versus measured overall performance (OP) (**b**). Pearsons’ correlations ($r_\textrm{p}$) and Spearmans’ rank correlations ($r_\textrm{s}$) are provided, the lines denote a linear regression fit

When predicting the breeders’ selections for individual year-site BLUEs, the performance was varying strongly (Fig. 4a). Clearly, breeders decisions were more related to a performance in an ‘optimal’ environment than to a low-yielding year-site. Keeping in mind that the F7 generation breeding experiment was actually performed in a low-yielding year-site (Delley, 2020), this capacity for abstraction is impressive: The prediction accuracy was highest for Delley, 2019, a year-site with high yield (Fig. 3a) and strong genetic correlations for common G$\times$E interactions with other year-sites (Fig. 3b). The lowest prediction accuracy was found for the year-site Delley, 2020, a year-site with below-average yield and lower genetic correlations for common G$\times$E interactions to other year-sites. When predicting breeders’ selections based on overall HTFP BLUPs with measured OP, the predictor performed better than for the best year-site (Fig. 4b).

**Fig. 5:**
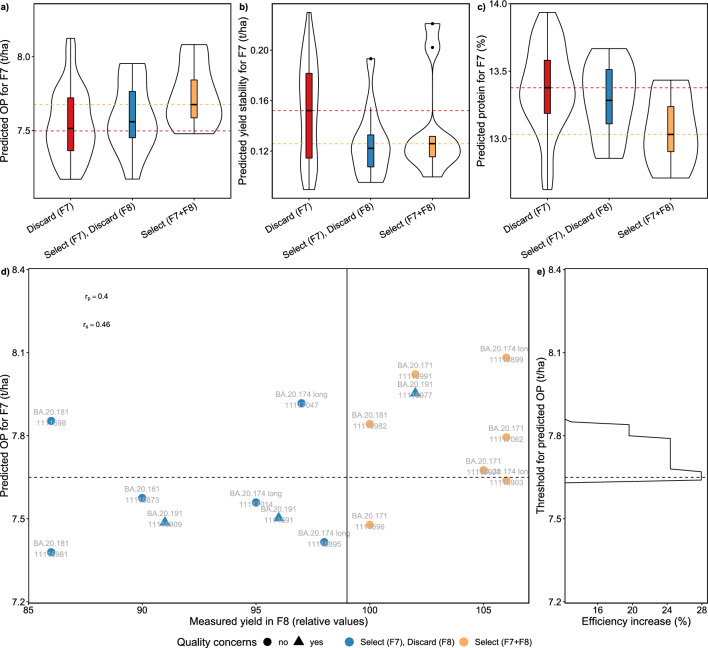
Predictions for F7 single lines for overall performance (OP) (**a**), yield stability (indicated by a low root-mean-square deviation value) (**b**) and grain protein content (**c**), predictions of OP versus measured yield for the subset of F7 lines that were selected and grown on yield plots in the subsequent generation (F8) (**d**), and efficiency increase if a phenomic selection thresholds would have been applied to the breeding program in F7 (**e**)

The consensus of OP predictions and breeders’ selections became further visible when predicting OP on single lines of the breeding experiment (Fig. 5a). The median predicted OP of discarded lines was clearly lower than the one for lines that were selected in the F7 and F8 experiments. Lines that were discarded after F8 were in-between the two extremes. Breeders avoided lines that had a low OP prediction for their selection. Nevertheless, some lines with above-average OP prediction were discarded.

When predicting yield stability (RMSD), medians of selected and discarded lines were close (Fig. 5b). Nevertheless, breeders avoided lines with high RMSD in the F7 and F8 experiments, with three exceptions. Again, some lines with above-average stability predictions were discarded.

Predicting grain protein content for single lines in the breeding experiment revealed a lower median grain protein content for selected lines than for discarded lines (Fig. 5c). The tendency for low grain protein content was also visible for lines selected in the F7 experiment but discarded in the F8 experiment.

When comparing predicted overall performance with yield measured in the F8 experiment, a clear linear relation for most lines became visible, resulting in an accuracy of $r_\textrm{s}=0.46$ (Fig. 5d). Applying an overall yield prediction threshold that maximizes the efficiency ($\sim$ 7.7 t/ha, Fig. 5) to the F7 selection experiment would have excluded eight genotypes with low yield predictions for the F8. For six genotypes, the low-yield prediction was confirmed in the F8, but two genotypes would have been excluded by mistake (Fig. 5d). In summary, the increase in efficiency if enhancing breeders selection decisions with a HTFP-based prediction threshold would have been in the range of 24–28% (Fig. 5e).

Predicting OP, yield stability and grain protein content for the selected F8 lines and F7 lines that were—despite their positive breeders’ ratings—not selected revealed no clear relation of OP versus yield stability (Fig. 7a). This result is unexpected, as it is commonly assumed that high-yielding genotypes cannot reach their full potential in every environment, thus decreasing stability. In contrast, the expected antagonistic nature of OP versus grain protein content was confirmed (Fig. 7b). Calculating the total harvestable protein per area based on these predictions revealed that selecting for high protein will inevitably reduce the harvestable total protein per area (Fig. 7b, grey lines).

### Breeders select differently than HTFP predictors

**Fig. 6:**
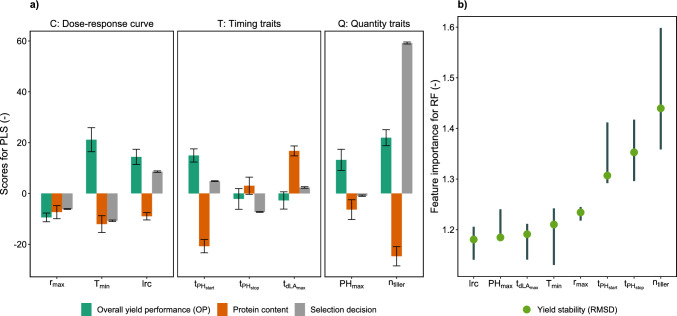
Scores for intermediate HTFP traits for the overall performance (OP) predictor, grain protein content predictor, and breeders’ selections predictors based on partial least squares (PLS) (**a**) and for yield stability (RMSD) predictor based on random forest (RF). Error bares in **a** are 95% confidence intervals based on jackknife variance estimates, in **b** 95% confidence intervals based on 50-fold cross-validation runs

Extracting PLS scores and RF feature importance of intermediate traits revealed that predictors trained on breeders’ selections and on OP focus on different trait categories and traits (Fig. 6). The score for HTFP traits that could explain breeders’ selections was highest and positive for $n_{\text{tiller}}$, followed by a negative score for $T_{\text{min}}$, a positive score for lrc, and a negative score for $t_{{\text{PH}}_{\text{stop}}}$. Consequently, traits of all three categories were among the important ones, while the most important one was a quantity trait (Q).

For overall yield performance (OP), the highest score was also found for $n_{\text{tiller}}$, but the score was less than half the size than for the most important one for the breeders’ selections. The positive score for the quantity trait $n_{\text{tiller}}$ was shorty followed by a similarly high score for the dose–response curve parameter $T_{\text{min}}$, for the timing trait $t_{{\text{PH}}_{{\text{start}}}}$, and the dose–response curve parameter lrc. Notably, in opposite to the breeders’ selection, the score for $T_{\text{min}}$ was positive.

Scores for grain protein content were to a large extent the opposite of scores for OP and/or yield stability. The largest and negative score was found for $n_{\text{tiller}}$, followed by a negative score for $t_{{\text{PH}}_{\text{start}}}$.

Feature importance values for yield stability were highest for the quantity trait $n_{\text{tiller}}$, followed by the two timing traits $t_{{\text{PH}}_{{\text{start}}}}$ and $t_{{\text{PH}}_{{\text{stop}}}}$. The feature importance of other traits ranged lower, but overall, differences were small, indicating that a nonlinear combination of all traits was required to predict yield stability.

Selecting for OP and yield stability simultaneously requires traits that are not antagonistic for those target traits. Such relations were found for the C trait $T_{\text{min}}$ where the absolute score for protein content was by a factor of 1.8 lower than the absolute score for OP. For the T traits $t_{{\text{PH}}_{{\text{start}}}}$ and $t_{d{\text{LA}}_{{\text{max}}}},$ the opposite was observed, having lower absolute scores for OP than for protein content by a factor of 1.4, respectively, 6.1. For Q traits, no promising candidates were found.

**Fig. 7:**
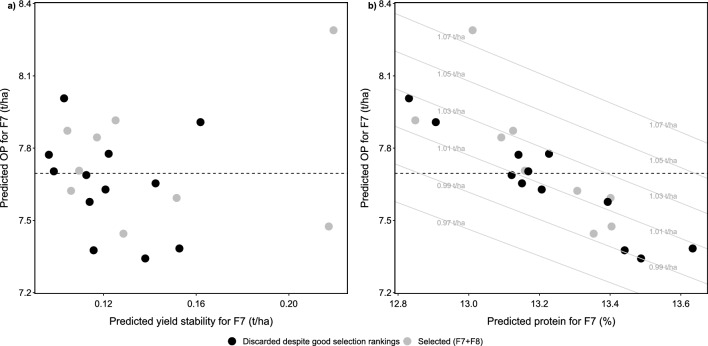
Overall yield performance (OP) predictions versus yield stability (indicated by a low root-mean-square deviation value) predictions (**a**) and versus grain protein content predictions (**b**) for the selected F8 lines (gray circles) and for possible candidates proposed by phenomic selection that were not considered (black circles). Numbers on **a**, **b** denote same genotypes, the black dashed line the proposed HTFP selection threshold for OP, grey lines and numbers in **b** denote equidistant protein per hectare lines

## Discussion

### The suitability of HTFP traits to predict overall yield performance, yield stability and grain protein content

A prerequisite for the indirect selection in breeding experiments is a strong relation of secondary traits to target traits. In this work, it was hypothesized and confirmed that for a subset of the examined HTFP traits, G×E interactions are less complex than for target traits, allowing an efficient selection in early breeding experiments. The low G×E interaction of HTFP intermediate traits may be reasoned by their close direct relation to the mechanistics of growth. In Roth et al. (2021), we elaborated that in particular dose–response curve traits (Category C) are less affected by G×E, as they can be seen as the driver of G$\times$E themselves. Indeed, for the five examined year-sites of this study, all three dose–response curve traits showed simple G$\times$E interactions without crossovers.

In contrast, for two out of four quantity traits (Category Q) more complex G$\times$E interactions were observed, indicating their limited robustness as environment-independent predictors for a target trait. Q traits may be regarded as area-under-the-curve representatives of growth processes (Roth et al. 2021) that are characterized by T and C traits. G$\times$E may increasingly build up during such a growth process. Consequently, a timing trait (Category T) related to one of these category Q trait, $t_{{\text{LA}}_{\text{max}}}$, showed more complex G$\times$E effects as well and had to be excluded.

Unlike the intermediate traits, the target trait yield showed partly complex G$\times$E interactions and could be separated in overall yield performance and yield stability. Wheat yield is a trait that can be dissected in physical components, namely, number of plants per square meter, number of shoots with fertile ears per plant, and number and weight of grains per ear (Stern and Kirby 1979). Each of these components is the result of dynamic growth processes that are driven by genetically determined plant demands and environment supplies (Triboi et al. 2006). Consistently, traits of all three categories were useful predictors of overall yield performance.

For grain protein content, early-season Q and T traits were the most useful predictors. N accumulation in grains is known to be source determined (Martre et al. 2003). Consequently, genotypic variation in grain protein arises mainly from differences in N utilization efficiency and harvest index (Le Gouis et al. 2000). Hence, if N is not a limiting factor, genotypic differences may be very small, and increasing yield will lead to decreased protein content due to dilution. Consistent with this, most intermediate HTFP traits responsible for high protein content were also associated with low overall yield potential. The most influential trait $n_{\text{tiller}}$ might hint on a dilution by an early development of a high number of ear-bearing culms. To elucidate such effects will require ear counts per unit area, a trait which caught a large interest in the field phenotyping community (David et al. 2021; Dandrifosse et al. 2022).

Additional contrasts for protein content may arise from an adapted growth duration (Triboi et al. 2006). Consistent with this hypothesis, T traits were important as predictors for grain protein content as well. In particular, an early start of the generative phase ($t_{{\text{PH}}_{{\text{start}}}}$) and a delayed maximum growth rate of apparent leaf area timing trait ($t_{d{\text{LA}}_{{\text{max}}}}$) showed high scores for the PLS predictor, reinforcing the finding that changes in phenology were among the main drivers of grain protein content differences.

### Formulating an ideotype

As elaborated above, yield and grain protein content are negatively related, to a large part explained by a dilution of protein by accumulated starch of high-yielding varieties. Despite these strong relations between traits, three intermediate traits were identified that allow optimizing for protein content as well as overall performance: From the C traits, the base temperature of growth $T_{{\text{min}}}$, and from the T traits the time point traits $t_{{\text{PH}}_{{\text{start}}}}$ where the stem elongation starts and $t_{d{\text{LA}}_{{\text{max}}}}$ where the maximum increase rate in apparent leaf area is reached.

**Fig. 8:**
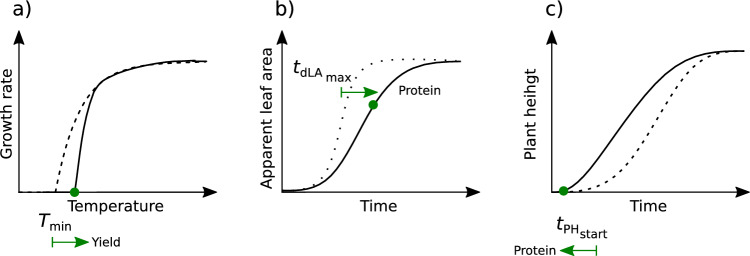
Proposed ideotype to select for high overall yield performance, yield stability and grain protein content based on dose–response curve traits related to the height development of the canopy (**a**) and timing of key stages traits based on apparent leaf area (**b**) and plant height (**c**). The optimization direction of traits is indicated with green arrows

The most promising ideotype that optimizes overall yield performance and grain protein content requires selecting for high values for $T_{{\text{min}}}$ to increase yield, and an early $t_{{\text{PH}}_{{\text{start}}}}$ to increase protein (Fig. 8a, c). As those two traits are genetically uncorrelated (Fig. 2), an independent selection may be easy to achieve. Indeed, in Roth et al. (2022b) we found evidence that for the Swiss varieties such an optimization has already happened. The reported relations of C, T and Q traits of this study are in accordance with the ones reported in Roth et al. (2022b), with one exception: While for the diverse GABI wheat panel examined in Roth et al. (2022b), $T_{{\text{min}}}$ was closely related to the phenology traits $t_{{\text{PH}}_{\text{start}}}$ and $t_{{\text{PH}}_{\text{stop}}}$, such a relation was not visible for the Swiss elite cultivar set examined in this work. Consequently, the observed effects in the GABI wheat panel may be partly related to population effects, further confirming the possibility for independent selection of $T_{{\text{min}}}$ and $t_{{\text{PH}}_{{\text{start}}}}$.

Alternatively, one could also select for a delayed development of apparent leaf area ($t_{d{\text{LA}}_{{\text{max}}}}$) to increase protein content (Fig. 8b). Interestingly, a similar ideotype was proposed based on growth dynamics’ HTFP traits in soybean (Roth et al. 2022a). Nevertheless, delaying canopy development in the late season requires special care: In this work, data collection was based on RGB imagery. Delayed T traits for apparent leaf area ($t_{d{\text{LA}}_{{\text{max}}}}$) may indicate an advantage of ‘stay-green’ genotypes (Thomas and Smart 1993). When formulating a corresponding ideotype concept, separating functional from ‘cosmetic’ stay-green is essential—only if canopies remain highly productive in such a stay-green phase a delayed senescence is beneficial. Consequently, if including such traits in a PS approach, one may have to extend the RGB phenotyping technology and include spectral measurements to monitor the dynamics of senescence (Anderegg et al. 2020) and thermal data to extract an indicator for functional stay-green (Anderegg et al. 2021).

### Enhancing breeders’ selections with HTFP traits

Training a PLS with breeders’ selection decisions and predicting year-site specific yield revealed that breeders select for an average performance for their TPE. Notably, the training set was based on selection decisions taken on a year-site that showed lower-than-average genetic correlations for common G$\times$E interactions to other year-sites. Consequently, the year-site was unfavorable for breeders as selection base. Nevertheless, the breeders managed to select in a year-site ‘neutral’ manner for overall performance, indicating that their selection was based on robust secondary traits with little G$\times$E interaction.

In opposite to overall yield performance, grain protein content was a trait on which selection does not seem to have a strong effect. While most of the traits showed converse effects on protein content and yield, some had a stronger effect on either of both. While an indirect selection for a high grain-protein deviation based on such information may be difficult, the information how intermediate traits affect these target traits is of major importance for breeders. Early vigor for weed suppression for example is increasingly important due to the political pressure for herbicide-free cropping systems. Thus, an increased vigorous development to achieve a better weed suppression in spring may reduce protein content while increasing yield.

For yield stability, breeders’ selections were neutral, they did not neglect but also not strongly selected for more stable genotypes. Again, with HTFP, breeders selection decisions could become enhanced with a stronger focus on stability. However, quantifying yield stability will need many test environments and genotypes. The 5 year-sites included in the present study offer only a limited insight into the driving factors of G$\times$E. Implementing HTFP in a lager number of sites including future climate sites may greatly enhance our understanding about ideotypes with a strong resilience to extreme weather conditions.

Introducing a threshold based on overall yield performance predictions with HTFP traits could increase the efficiency of selection by up to 28%. Predictions for the discarded lines in the F7 breeding experiment made evident that potential candidates with high yield and protein potential were present in the breeding program. The prediction accuracy ($r\approx 0.4$) of PS trained on a small set (45 genotypes) was comparable to those measured in GS approaches with large training sets ($>550$ genotypes) (Rutkoski et al. 2016; Crain et al. 2018; Sandhu et al. 2021). If keeping all other parameters of a breeding program constant, such an efficiency increase would directly translate into genetic gain, making PS for the particular case as competitive as GS. The achieved rank prediction accuracy of unseen genotypes in an unseen environment of 0.46 further underpins this finding.

In addition, PS and GS do not necessarily have to be seen as competing—integrative approaches may also be conceivable. Diepenbrock et al. (2021) for example used GS to predict parameters of a crop growth model that related to the target trait yield, which improved the accuracy of yield predictions for unseen genotypes in unseen environments. In this work, it was demonstrated that if the TPE is part of the PS training set, the non-complex G$\times$E nature of intermediate HTFP traits may allow comparable target trait prediction approaches without explicitly including environmental covariates. In return, this could potentially allow predicting intermediate traits directly with GS instead of measuring them with HTFP.

Once trained, the presented phenomic selection approach can be applied to single-row plots in early generations. Nevertheless, the training set requires to be collected in generations grown in yield plots. This study was conducted within the framework of the Swiss winter wheat breeding program of Agroscope/Delley Samen und Pflanzen AG, without interfering with it. Starting with the F8 generation, yield plots are used in this breeding program. The F8 generation is grown in 1 year only, and most of the examined $\sim$ 200 genotypes discarded afterward. An essential fundamental of the presented approach is to examine G$\times$E interactions of target traits (yield, grain protein content) and high-throughput phenotyping traits. As source for G$\times$E interactions in wheat cultivation in Switzerland, the year is as important as the location (Fig. 3b). Using the F8 generation as training would have prevented from examining year-based G$\times$E interactions, therefore limiting the power of the training set. Consequently, it was taken advantage of the variety testing character of the subsequent F9–F12 generation experiments that are performed in multiple years. Depending on the breeding program, the generation that is best suited as training and selection set may vary. Importantly, the training set has to be as genetically close to the selection set as possible, while providing sufficient reliable yield measurements in multiple locations and years. For the Swiss winter wheat breeding program, this prerequisite is fulfilled in the F9–F12 generation experiments.

### Considerations on the added value of PS versus GS to support the Swiss winter wheat breeding program

The attractiveness of a particular approach for a breeding program depends to a large extent on the costs. In the following, the economical costs and accuracy increase of PS versus GS is evaluated on the case example of the Swiss winter wheat breeding program of Agroscope (Nyon, Switzerland)/Delley Samen und Pflanzen AG (Delley, Switzerland). A base assumption is that the design of the program itself remains unchanged and that GS and PS could be introduced independently at certain breeding stages. Cost estimations for GS are based on know-how gained during the implementation of GS in the Agroscope/Delley Samen und Pflanzen AG breeding program. Cost estimations of PS are based on annual costs of the Trait Spotting project where two sites per year with a total area of 2.160 m$^{2}$ were monitored.

Given is the winter wheat breeding program with 300,000, 12,500, 5000, 2500, 900, 220, 60, 30, 36, and 36 genotypes in the F3–F12 generations (Fig. 8a). Given is further that F3 are single plant, F4 single rows, F5 double row plots, F6 four-row plots, F7 20-row plots, and F8–F12 yield plots, while rows have a 0.25 $\times$ 1 m and yield plots a 1.5 $\times$ 5 m area (Fig. 8b). Finally, given is that for F3–F6 one location, for F7 two locations, for F8 four locations, and for F9–F12 five locations are used (Fig. 8a). The measured accuracy for yield prediction with PS in this work was $r=0.43$, for measuring yield with yield plots $r=0.9$, and the provided accuracy for GS in wheat is $r=0.32$ (Crain et al. 2018).

**Fig. 9:**
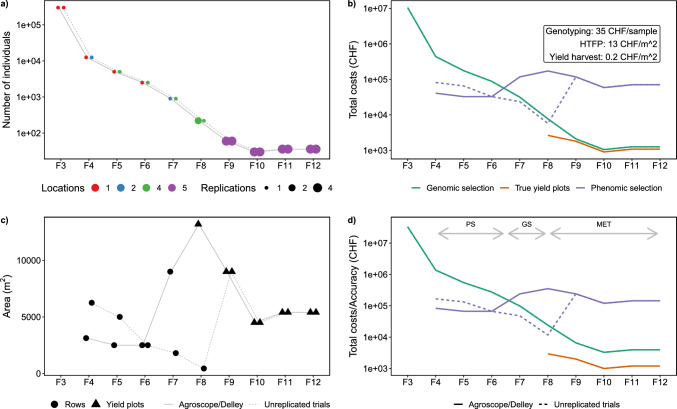
Dimensions of the Swiss winter wheat breeding program and an alternative program with unreplicated trials per location (**a**, **c**) and costs (**b**) versus accuracy (**d**) of a genomic selection approach (GS) based on genotyping and a phenomic selection approach (PS) based on high-throughput field phenotyping (HTFP) and classical yield multi-environment trials (MET)

When using these values to calculate total costs per breeding stage, costs for GS in early breeding stages (F3–F6) are higher than for PS, as the number of required samples are high but the areas to monitor small (Fig. 9b). Starting with F7, GS becomes more affordable than PS. Finally, starting with F8, yield plots can be used, representing the most cost-efficient selection strategy. The choice on replicated or unreplicated trials per location may influence these findings: If distributing the available rows per generation in the Swiss breeding program to unreplicated trials at many locations instead of replicated trials at a few locations, PS may be more affordable than GS in later generations (Fig. 9). Nevertheless, such an approach would most probably require the use of double-haploids to ensure the genetic stability. Still, the fact that phenomic data in a breeding program is often collected for other reasons as well gives PS further justification.

Given that both GS and PS are not yet fully part of a breeding program yet, breeders would be more interested in the return in accuracy increase if adapting new technologies than in total costs. When dividing the total costs by the expected selection accuracy, costs of increasing the accuracy of selection are lowest for PS in early breeding stages, lowest for GS or PS in intermediate breeding stages (depending on whether locations include replicated trials), and lowest for yield trials in late breeding stages (Fig. 9d). In summary, for the Swiss winter wheat breeding program, a hybrid GS/PS strategy may be best suited to increase the genetic gain, with PS focusing on early single and double-row plots.

## Conclusion

Using HTFP to extract dynamic traits of three different categories (C, T, Q) derived from drone-based RGB imaging proved to be suitable to allow a prediction of overall yield performance, yield stability, and grain protein content. Less complex G$\times$E interactions of HTFP traits than those of target traits allowed the application of HTFP even in early breeding generations with few locations. Traits related to quantities at defined time points or time periods (Category Q) can be seen as proxy traits for yield components and are the result of related growth processes, giving them high prediction potential for overall yield performance and protein content. Timing of key stages (Category T) and dose–response curve (Category C) traits are more related to stability. This suggests focusing on such traits when aiming at the mitigation of the effect of abiotic stresses. As dose–response curve traits have lowest G$\times$E interactions, they potentially allow performing predictions on HTFP data from single locations.

The prediction accuracy of phenomic selection (PS) is comparable with that reported for genomic selection (GS) and allows increasing the selection efficiency by up to 28%. While costs of GS mainly depend on the number of genotypes to sample and continuously decrease with increasing generations, PS depends on the area to monitor, making it cost-efficient for early breeding stages. Other than GS, PS allows analyzing the influence of dynamic traits on target traits, providing both insights on existing breeders’ selection processes but also formulating an ideotype concept for further refinements of those. In particular, the C traits base temperature of growth, and the T traits start of stem elongation and time point of maximum apparent leaf area increase allowed formulating an ideotype concept that may optimize yield, yield stability and protein content.

## Supplementary Information

Below is the link to the electronic supplementary material.

Supplementary file 1 (pdf 372 KB)

## Data Availability

The datasets generated and analyzed during the current study are openly available in the ETH Research Collection repository, http://doi.org/10.3929/ethz-b-000566864. Source code for the phenomics data processing methods used in this study are openly available in the ETH gitlab repository, https://gitlab.ethz.ch/crop_phenotyping/htfp_data_processing.

## Footnotes

1 Please note our consciously chosen relaxed definition of phenomic selection (PS) in contrast to Rincent et al. (2018) and Robert et al. (2022): While Rincent et al. (2018) restricted PS to depend on similar statistical models as GS, Robert et al. (2022) went one step further and defined the input of PS to be based on near-infrared (NIR) spectroscopy data. Unlike NIRs, however, most phenomics’ techniques allow resolving environmental-specific phenotypes, calling for other statistical models than GS, and widening the scope of PS far beyond NIRs data.
