# Supplementary material for: Image-based phenomic prediction can provide valuable decision support in wheat breeding
Source: Theor Appl Genet. 2023 Jun 27;136(7):162. doi: 10.1007/s00122-023-04395-x (PMC10299972; doi:10.1007/s00122-023-04395-x)
Supplement: Supplementary file 1 — (pdf 372 KB) [file 122_2023_4395_MOESM1_ESM.pdf]

Appendix

Figures

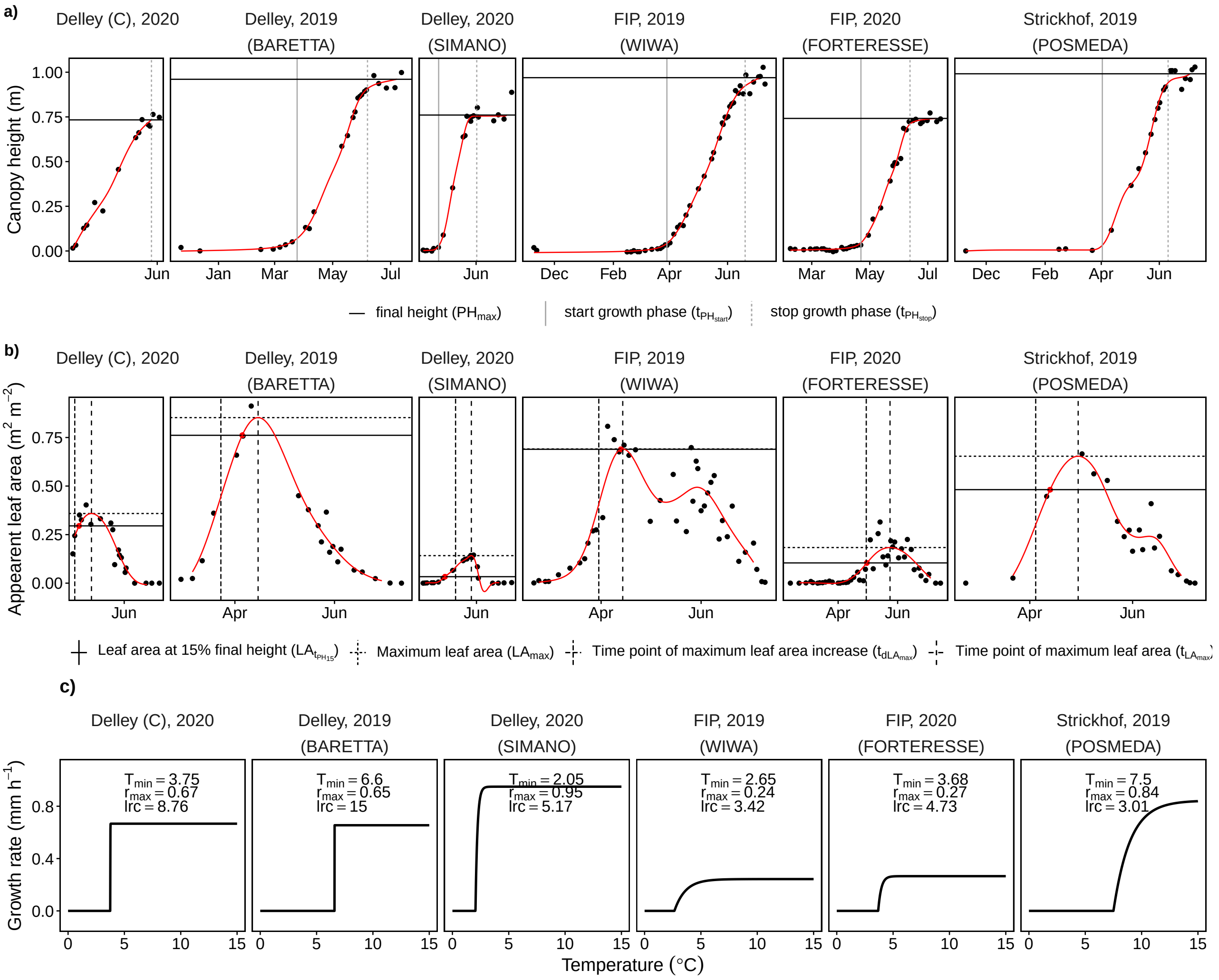

Figure S1: Examples of extracted low-level traits canopy height (a) and apparent leaf area (b) with measured time points (black circles) and fitted P-splines (red lines) and fitted temperature dose-response curves (c) for six plots of six year-sites.

Site Delley

Delley19 (Genevey 2):

The site Delley 2019 was located at Delley, Switzerland (46.918 N, 6.979 E, 500 m a.s.l.). The soil had a clay content of 15-25%, humus content was 1.5% and pH 7.2. Soil characteristic were determined in 2017 (Sol Conseil, Nyon, Switzerland). Preliminary to wheat, pea (*Pisum sativum* L.) were grown. After preliminary crops were harvested, the soil was irrigated with 20 mm water, plowed and harrowed before wheat was drill-sown.

For the design, varieties were arranged in a 6 x 6 lattice design with four replications. The experimental unit was a plot of 9 m^2 (1.5 m in row and 6 m in range direction including paths and wheal tracks), resulting in a harvested surface of 7.05 m^2.

Wheat was drill-sown in 8 rows per plot with a row distance of 0.125 m on October 24, 2018. Sowing density was 350 plants m^{-2}.

On February 25 and April 1, 84 kg N $^{-1}$ and 65 kg N $^{-1}$ ha^{-1} were applied. Additionally, in total 50 kg phosphorous ha^{-1}, 75 kg potassium ha^{-1}, 20 kg calcium ha^{-1} and 18 kg magnesium ha^{-1} were applied.

Delley20 (Grandcour):

The site Delley 2020 was located at Gletterens, Switzerland (46.88971 N, 6.93694 E, 482 m a.s.l.).

Soil characteristics: Clay content: 20%, pH: 6.4, humus content: 1.6 (Sol Conseil 2022, Nyon, Switzerland)

Preliminary crop was clover, followed by potatoes.

For the design, varieties were arranged in a 6 x 6 lattice design with four replications. The experimental unit was a plot of 9 m^2 (1.5 m in row and 6 m in range direction including paths and wheal tracks), resulting in a harvested surface of 7.05 m^2.

Wheat was drill-sown in 8 rows per plot with a row distance of 0.125 m on November 12, 2019. Sowing density was 350 plants m^{-2}.

Additional management information:

20.01.2020 165 kg/ha Landor 0/20/30
17.03.2020 368 kg/ha Nitrate magnésien soufré 24/0/0 (88 kg N/ha)
18.03.2020 0.5 kg/ha Artist + 1 l/ha Netzmittel + 200 g/ha Othello Star
04.05.2020 294 kg Sulfonitrate 26/0/0 (77 kg N/ha)
27.02.2020 Harvest
(Total 165 kg N/ha)

Site FIP

The site FIP is located at the ETH research station of agricultural sciences in Lindau Eschikon, Switzerland (47.449 N, 8.682 E, 556 m a.s.l.). The soil type is an eutric cambisol consisting of 21% clay and 21% silt. Organic matter content is 3.5% and pH 6.7. Soil characteristic were determined in 2015 (Eric Schweizer AG, Thun, Switzerland). Preliminary to wheat (*Triticum aestivum L.*), soybeans (*Glycine max* (L.) Merr.) and buckwheat (*Fagopyrum esculentum* Moench) were grown. After preliminary crops were harvested, the soil was plowed and harrowed before wheat was drill-sown.

FIP19:
For the design, 36 test varieties were arranged in four replications equally allocated across two lots of the FIP. The experimental unit was a plot of 6.1 m^2 (1.5 m in row and 6 m in range direction including paths and wheal tracks). The varieties were allocated in a row-column design as follows: Full replicates in row direction consisted of six rows by six ranges. Blocks in range direction consisted in two ranges spanning the 24 rows of both lots, thus holding 1.33 replications per genotype. This dimension was introduced to cover the spatial trend of the sloped field in an upper, central and lower part. The design was generated using the R-package DiGGER.

Wheat was sown in 9 rows per plot with a row length of 5 m and a row distance of 0.125 m on October 17, 2018. Sowing density was 400 plants m^{-2}. One day after sowing, herbicide (Herold SC, Bayer AG, Leverkusen, Germany) was applied to ensure weed free plots. Several fungicides and insecticides were applied in spring to ensure healthy plants.

On February 27, 2019, April 8 and May 27, 52 kg N $^{-1}$, 72 kg N $^{-1}$ and 24 kg N ha^{-1} were applied. Additionally, in total 92 kg phosphorous ha^{-1}, 120 kg potassium ha^{-1} and 15 kg magnesium ha^{-1} were applied.

FIP20:
For the design, 36 test varieties were arranged in three replications on one lot of the FIP. The experimental unit was a plot of 6.1 m^2 (1.5 m in row and 6 m in range direction including paths and wheal tracks). The varieties were allocated in a row-column design as follows: Full replicates in row direction consisted of six rows by six ranges. Blocks in range direction consisted in two ranges spanning the 18 rows of the lot. This dimension was introduced to cover the spatial trend of the sloped field in an upper, central and lower part. The design was generated using the R-package DiGGER.

Wheat was sown in 9 rows per plot with a row length of 5 m and a row distance of 0.125 m on October 17, 2019. Sowing density was 400 plants m^{-2}. Eight days after sowing, herbicide (Herold SC, Bayer AG, Leverkusen, Germany) was applied to ensure weed free plots. Several fungicides and insecticides were applied in spring to ensure healthy plants.

On March 3, 2020, 26 kg N ha^{-1} and 58 kg Mg ha^{-1} were applied. On April 27, 2020, 72 kg ha^{-1} and 15 kg Mg ha^{-1} were applied. On May 7, 2020, 24 kg N $^{-1}$ and 5 kg Mg ha^{-1} were applied.

Site Strickhof

The site Strickhof is located at the Strickhof Landwirtschaftsschule in Lindau Eschikon, Switzerland (47.445 N, 8.683 E, 535 m a.s.l.). The soil was a skeleton rich Cambisol (Landwirtschaftsamt des Kanon Zürich, 1992) with 34% clay, 3.3% organic matter and a pH of 7.4 (Martin Bertschi, Strickhof, personal correspondence, October 2019).

Strickhof19:
There were four replications on one lot at the Strickhof site where the genotypes were arranged in a random complete block design. The management was less intensive, following the guidelines of Federal Swiss pesticide reduction program "Extenso". No insecticides, fungicides or plant growth regulators were applied. Fertilization regime was following common agricultural practice. Sowing took place on October 17, 2018, with the same specifications as at ETH-FIP site but at a length of about 6.4 m.
